# Abstracts &c.

**Published:** 1889-05

**Authors:** 


### ABSTRACTS AND GLEANINGS.

Mr. Fleming, M. R. C. S., says in *British Med. Jour.:* We want a surgical dressing that is non-irritating, antiseptic, will not become adherent, will allow free drainage, will not allow the discharges to get hard and caked, will be truly miscible with discharges, and not evaporate at any temperature of the body, nor occupy the place intended for the discharges.

He thinks we have the disideratum in the glycerite of starch, with some antiseptic dissolved in it, e. g., corrosive sublimate, 1 in 1,000 parts. This application is not irritating, is antiseptic, is removed easily from any wounded surface, and fulfils, more nearly than any other, all the requirements above named. It is most commonly applied thickly spread on one or more layers of gamgee tissue, or some other absorbent tissue. Mr. F. has had good results with it, as far as he has given trial.—*Popular Science News.*

**Hernia.**—Dr. Perro (*Centralbl. fur Chirurg.*) recommends the following simple procedure for reducing strangulated inguinal hernia. The pelvis is raised by a cushion. and the leg flexed and abducted. The scrotum and hernial tumor are grasped by the left hand, and the tumor slightly bent toward the abdominal wall and compressed. At the same time, the index finger of the right hand is introduced into the inguinal canal, and, by a boring movement, pressed in the direction of the horizontal ramus of the pubes. In a short time, the strangulated portion of the intestine slips into the abdominal cavity, and is followed by a disappearance of the tumor. The author has succeeded in this manner in reducing six cases of strangulated hernia, when all previous attempts had failed.—*Ib.*

**How Vaccination Takes with the Indians.**—Official information having recently been received by Dr. Benjamin Lee, Secretary of the State Board of Health, from the Secretary of the New York State Board, of the existence of small-pox among the Indians of the Cataraugus and Allegheny Reservations in that State, he at once telegraphed instructions to Dr. J. L. Stewart, of Erie, Medical Inspector to the Board for the Lake District, to visit the Cornplanter tribe, in Warren county—the members of which are in constant communication with those of the New York reservations—to take the necessary means for their protection. The instructions were carried out with commendable promptness. The entire tribe was vaccinated, and the chief, Marsh Pierce, promised to forbid his people from crossing the border until the disease disappeared. He rendered every assistance to the vaccine physician, and extended the thanks of himself and his people to the State Board for its timely action in their behalf.—*Col. and Clin. Record.*

**Treatment of Hemorrhagic Malarial Fever.**—Dr. Mclaughlin, in New Orleans Medical Journal of March, 1889, remarks:

The treatment of hemorrhagic malarial fever is a subject of much dispute. Those who regard the disease as being of malarial origin give quinine in small, medium or large doses, according to the severity of the attack or the condition of the patient. On the other hand, many physicians, some of large experience, do not give quinine. In fact, they regard it as being harmful in the treatment of this disease. Others, who, in their early experience, had given quinine, were induced to abandon it from the bad results which they obtained from its employment, and claim that since they have abandoned quinine in the treatment of this disease a larger proportion of their cases have recovered. I have examined all the literature which I could obtain bearing upon the treatment of this disease, in order to ascertain to what extent this objection to the use of quinine prevailed, and, if possible, upon what grounds it rested.

A review of 640 cases, reported by forty physicians, compiled by Dr. Jerome Cochran; 268 cases, which serve as the basis of Ferand's study; 33 cases reported by Dr. R. D. Webb; 140 cases reported by Dr. G. B. Malone; in all 1,081 cases, to say nothing of isolated cases reported in various medical journals, indicate that a large majority of the physicians who reported the cases advise the use of quinine in the treatment of this disease; those who oppose it do so from the belief that it increases or precipitates the hæmaturia, and that the disease is better and more successfully treated without quinine.

Dr. McDaniel of Camden, Ala., says, with reference to this matter: "Now, my professional brethren, I know that I here step upon awful, solemn, dangerous and responsible ground, and being in general, as you all know, an advocate of quinine to an extent that few prudent men can claim to exceed, I take the position that I do here, not without having made the proper, thoughtful and conscientious pause. In Dr. East's experience, as I read it to you, and in much more that I did not read to you, just as often as he got his patients into a satisfactory condition and commenced giving quinine, just so often he plunged them back into hematuria, with its unnumbered woes and dangers." * * * "And over and over again have I, in my own practice, observed the hematuria re-established or aggravated after quinine; and, on the other hand, have seen the disease, with a very formidable array of symptoms, go handsomely on, without a grain of quinine, to a happy convalescence."

Dr. Webb says: "Quinine will undoubtedly, in a certain number of cases, increase the hematuria, and sometimes even seems to cause it.

Seeing this, the timid administrator stops his quinine, and his patient dies, with the quinine under the ban of killing him. Whereas, a bolder hand, directed by a proper idea of the true cause of this symptom, would have unhesitatingly continued it, and his patient might have had a good chance to live."

In 93 cases treated by Dr. McDaniel without quinine 16 died—a ratio of 10 per cent.

In 23 cases treated by Dr. Webb with large doses of quinine only 2 died—a ratio of 8.6 per cent.

In 73 cases treated by Ferand with large doses of quinine 5 died—a ratio of 8.6 per cent.

In 71 cases treated by the same author with very small doses of quinine 22 died—or a ratio of 31 per cent."

Whilst these figures prove that recovery may take place without quinine, they conclusively prove that quinine is not a dangerous remedy in this disease; on the contrary, the best results were obtained in those cases where quinine was given in large doses. Many physicians prefer the hypodermic method of giving quinine. The effects are more certainly obtained, whilst the nausea, which its bitter taste is so apt to excite, is avoided by this method. In many of the cases which I treated in 1868–9 quinine was given hypodermically. I was first induced to adopt this method from reading a statement in the Journal of Applied Chemistry to the effect that the addition of bile to a solution of quinine resulted in the formation of sulphate of soda and chlorate of quinine, and that the latter salt was to a great extent insoluble. My experience at the time with the hypodermic use of quinine would cause me to again use it, especially in those cases where the stomach and bowels were filled with bilious matter. Less objection is made to the use of calomel in the treatment of hemorrhagic malarial fever. A very large majority of the authors whom I have consulted advise its use, generally in connection with bicarbonate of soda; some in a free purgative dose or doses; others in smaller doses, to "stimulate the liver" or change its supposed abnormal action. Dr. McDaniel's treatment consists in giving calomel and soda, of each two grains, every two hours until constipation is overcome or diarrhœa corrected. He advises pellets of ice to relieve thirst and nausea, and external application of heat by means of vapor baths, etc. Ferand tried the treatment by calomel, both as purgative and alterative, and subsequently says: "I have arrived at the conclusion that calomel is far from having the efficacy that some suppose, and indeed that its employment is attended with serious disadvantages under many circumstances. I have therefore arrived at the point of rejecting it entirely in my practice."

Dr. Malone says: "Especially do I warn you against the use of calomel, quinine and turpentine." His treatment consists in giving large draughts of cool water, which he claims washes out the stomach, cools the fever, relieves the thirst and nausea, and is the best diuretic and sudorific. He strongly recommends that hyposulphite of soda be given in connection with fluid extract of buchu. Forty-four cases treated in this manner by Dr. Malone recovered without a single death. Ergot, muriated tincture of iron, atropine, gallic acid, etc., have been used and recommended, but correct data relating to his remedies is too meagre to form any opinion of their merits. My own experience in the treatment of this disease is that quinine is always indicated. I give it in free doses; and have never seen or known it to cause harm. It is my custom to precede its use in this, as in malarial fever, by one or more purgative doses of calomel, and am confident the physiological effects of the quinine are better obtained after the mercurial action. Nausea and vomiting call for the use of ice; small pieces of ice to be frequently taken. Failing in this, would direct that hot water be sipped or drunk freely by the patient. Sulphate of morphia, given hypodermically, will often quickly relieve the nausea, and at the same time will relieve the restlessness which is so annoying to the patient. This remedy, however, is not without its dangers, and should be used sparingly, or not at all, in those cases which show a tendency to anuria. Diuretics of a mild character, those which do not irritate the kidneys, are valuable remedies. I have found that lager beer is one of the most valuable remedies of this class. It is efficient as a diuretic, without irritating qualities, a good tonic, safe stimulant and fair hypnotic, and, above all, it is generally grateful to the patient and acceptable to the stomach when other remedies are not retained. Diaphoretics, especially when the action of the kidneys are sluggish and the skin is hot and dry, are, I think, necessary to a correct treatment of this malady. Warm or vapor baths, warm packs and the hypodermic use of muriate of pilocarpus are efficient agents. In the convalescent stages the continued use of quinine in small doses, with iron and perhaps strychnine, are demanded.

The manifest tendency to relapse, so characteristic of this disease, and frequently so fatal in its results, should never be forgotten. In conclusion, I will ask your attention to the diffuse nephritis, which is present in a large majority, if not all cases of this disease, and urge that this condition be not overlooked in our treatment. In a large majority of the fatal cases of hæmaturia death has resulted from suppression of urine. When this condition has existed for forty-eight hours, death almost invariably is the result; hence the importance of not overlooking the kidney lesions of this disease. Digitalis is advised when the pulse is feeble, rapid or dichrotic. Food and stimulants, when indicated by mouth or rectum, should be given in this disease, as in others, in such amounts and of such kinds as each individual case may require.

In conclusion, I submit the following report of two cases of hemorrhagic malarial fever which recently came under my care: On the 15th of last November I visited Mrs. S—, and met Dr. Weisselberg, the attending physician, in consultation. The doctor informed me that Mrs. S— had been under his care for several days past, and furnished the following history of her sickness. She had frequent attacks of malarial fever during the latter part of the autumn and fall; her husband had been similarly affected. Their home, in an adjoining county, is on a farm of new land, the most of which was cultivated this year for the first time. Malarial fever prevailed in their neighborhood to a much greater extent this year than at any former period for several years past. Her fever was of the secondary type, for which Dr. Weisselberg had been giving quinine quite freely, about forty grains during the intermission, without interrupting the paroxysms. The night preceding my visit she had an unusually hard chill, followed by high fever and delirium.

The following morning, at the time of my first visit, we found her with a temperature of 105 degrees Fahrenheit and delirious. She was six months pregnant; uterine contractions were quite marked, and the family informed us that she had previously complained of pain in her lower abdomen. As no urine had been passed by our patient during the previous night or up to the time of our visit, a catheter was introduced and we obtained about sixteen ounces of dark-red turbid urine, which had the color of porter. The following treatment was ordered: Calomel, grs. x, to be taken at once; ten grains of quinine every two hours; digitalis, m. 10, potassi bromidi, grains 10, every three hours. Lager beer, which was relished by the patient, was given as freely as desired. At our evening visit we found our patient with a temperature of 102 degrees Fahrenheit, with less delirium. The urine was passed in fair amount and continued bloody; the bowels had not acted.

The following morning our patient was without fever, mind clear; the bowels had moved freely during the night, and her urine was of normal color and amount. From this time she continued to improve. The intervals between doses of quinine were lengthened; the other medicines, except the beer, were discontinued.

I direct your attention to the following facts connected with the history of this case: The history of previously existing malarial diseases; the failure to control these by seemingly large doses of quinine, and the rapidity with which the disease yielded when the amount of quinine was increased, preceded, however, by the mercurial purgative. Another matter of interest is that under these larger doses of quinine the uterine contractions ceased and a threatened miscarriage was prevented.

I was called to see the second case Dec. 1st, 1888. The patient was a ten year old daughter of Mr. W., who lives about twenty miles distant, and had arrived in Austin the day before the date of my first visit. Her temperature was 103½ degrees Fahrenheit; pulse 140 dichrotic and feeble; skin was intensely jaundiced and dry. There existed mild delirium, thirst, restlessness, pain in the head and incessant nausea. She had a severe chill the night of her arrival in Austin. My first visit was made about noon on Dec. 1st. She had just passed about eight ounces of bright cherry-red urine. Early in the day I was informed that she had passed urine of a similar color. Mr. W., the father, informed me that his family had fever and bilious attacks quite frequently during the past summer and fall, and that the little patient particularly had been thus affected, for which he had repeatedly given her Simmons' liver medicine and quinine. They live in a very malarious section of country. The following treatment was ordered: Frequent sponging of the body with warm water, warm foot-baths and hot water tea to be used *ad libitum*. Ordered sol. magnesia citrate to move the bowels, and tr. digitalis gtt. v., liq. ammonia acet. ℥j, every three hours. At the time of my morning visit I found the condition of the patient unchanged. Her bowels had moved freely from the magnesia, but no urine had been passed since twelve o'clock the day before. Ordered—pilocarpus muriate gr. ⅛, tr. digitalis gtt. v., every two hours till free diaphoresis was produced; the former prescription to be discontinued; warm bath to be given at once, and five grains of quinine every three hours; if not retained, the amount to be doubled and given by enema.

The next morning her fever had gone; pulse 140, very feeble and dichrotic; no urine passed during the night or for eighteen hours; skin moist; less vomiting. The quinine ordered the night before was not retained by the stomach, except, perhaps, one dose, and, contrary to my instructions, the family had not given it by the bowels; gave at once twenty grains of quinine by enema, and ordered ten grains to be given in the same way every four hours. Ordered: Calomel, grains x; pulv. sugar of milk, grains xx, in two powders, one of them to be taken at once, the other in three hours if the bowels did not move. Continued the hot water tea, which was grateful to the little patient, who drank it freely; discontinued the beer, as it was not retained, and gave in its place whisky toddy. Hot poultices were applied to loins, and the mixture of pilocarpus and digitalis was continued. Saw her again at noon; no fever, pulse 110 with more force and not so thrilling; stomach quiet and nourishment taken very well; had passed about one ounce of clear urine; same treatment was continued. Evening visit—no fever, less restless, nausea relieved, profuse diaphoresis; had passed about one ounce of urine since noon visit, which was of normal color. As she was thoroughly cinchonized the doses of quinine were given every six hours; otherwise there was no change made in treatment.

At my next visit, the following morning, I found my little patient better in every respect. She had passed urine several times during the night, about an ounce of clear urine each time; had perspired freely, was less jaundiced, and manifested some desire for food. Ordered that three grains of quinine should be given every six hours for one week, also the following: ℞. Strychnine sulph. gr. ½; tr. ferri chloridi ℥j; M. sig. Ten drops in water three times a day. Mr. W. reported in the evening that his daughter continued to improve. He left next morning for his home.

**Cocaine in Dentition.**—M. Viguiere has proposed the following to relieve the pain which children suffer when cutting their teeth, especially the canine teeth: ℞.—Cocaine hydrochlorat., grs. 2; syrup. simpl., dr. 2½; Tinct. saffron, gtt. 10. M. Sig.—Rub the painful parts of the gums many times a day.—*La Chimique*.

**How to Preserve the Physician's Hands.**—The question how to disinfect the hands when a surgical operation is to be performed having been quite well settled, a problem now arises of how to preserve the hands against the assaults of modern antisepsis. The frequent washing, scrubbing, and contact with carbolic acid, corrosive sublimate, etc., sometimes ends in making the hands rough, sore, red and unsightly. At least this is the complaint of some of our German confreres to whom, if report speaks correctly, there have not descended hereditary protective influences from ancestral scrubbings. Dr. George Meyer, of Berlin, who has been himself a sufferer from "rothen Handen," describes a method of treatment which gives great relief. After washing the hands in soap and water, and carefully drying them, the following ointment is thoroughly rubbed in:

| | | |
|---|---|---|
| ℞. | Lanolin puris . . . . . . | 100.00. |
| | Vanillin . . . . . . . . | 0.1. |
| | Ol. rosar . . . . . . . | gt. j. |

M.

The vanillin and oil of rosemary are, of course, not essential. After the application the excess is wiped off upon a towel. Small tubes, like those used by artists for holding paints can be filled with the ointment, and it is thus made easily portable.—*N. Y. Med. Record.*

**Some of the Abuses of Etherization.**—Dr. George F. Shrady read a paper on this subject before the Practitioner's Society of New York, and concluded with the following: 1. In commencing the administration of ether the gradual method is to be preferred.

2. Its employment allows the lungs to empty themselves of residual air, prevents coughing and struggling, and places the organs in the best possible condition to receive and rapidly utilize the ether vapor.

3. After the stage of primary anæsthesia is reached, the more pure ether vapor the patient breathes the better.

4. The shorter the time of anæsthesia, and the smaller the amount of ether used, the less likely are the unpleasant sequelæ to occur.

5. The more evenly it is administered the less shock to the patient.

6. Anæsthesia should be entrusted to experienced administrators only.

7. Many of the fashionable efforts to resuscitate patients are not only useless but harmful.

8. The minimum amount of force should be employed to restrain the muscular movements of the patient.

9. Mixed narcosis is often advisable for prolonged operations.

10. The utility of the galvanic battery, in threatened death, is yet to be proven.

11. The most trustworthy means of resuscitating desperate cases are artificial respiration, hypodermic stimulation, inhalation of nitrite of amyl, and inversion of the body.

In the discussion of the paper above, Dr. Wm. T. Bull was strong in his denunciation of the rapid or drenching method of administering ether.

Dr. Joseph D. Bryant thought that the kind of cone to be selected was of much less importance than the proper employment of the one that was used. He referred to the fact that any disturbance of the patient, either by being handled or by the talking of those about him, always rendered the induction of anæsthesia more difficult. Everything should be quiet.

Dr. Samuel Sexton protested against the cruel manner in which children were often anæsthetized—the holding of the child's head forcibly against the table by pulling on the hair. He endorsed what the author said about forcible expiration to get rid of the residual air just as the ether is about to be given.

Dr. James B. Hunter agreed with Dr. Shrady in his condemnation of the custom of leaving the administration of ether to the Junior assistants of a hospital.—*Practice.*

**Sulphonal.**—More than a half dozen practitioners of this city were recently asked their experience with sulphonal. Up to time of going to press we have received replies from the following:

Dr. Hunter McGuire likes the remedy very much, and uses it frequently. He has found it particularly valuable in insomnia following the use of alcohol. Fifteen grains is the dose generally prescribed, and he has found that this should be largely diluted to get the best results. This quantity given in a tumblerful of milk at bedtime acts admirably well. One of his patients took, by mistake, sixty grains of sulphonal at one time. He slept for twenty-four hours, but could be easily aroused during this time to take nourishment—going off to sleep again. No bad effects were produced.

Dr. George Ross has also used sulphonal frequently, and in suitable cases he is pleased with the new hypnotic. He has not observed any unpleasant or deleterious effects. The minimum dose employed by him is fifteen grains, but twenty grains is more certain.—*Ib.*

**Magnesii Salicylas.**—Magnesium salicylate is a new antiseptic introduced by Huchard. It is met with in the form of long, colorless needles, has a bitter taste and is freely soluble in water and alcohol. It has been highly recommended in abdominal typhus as a superior substitute for salicylate of bismut; being less astringent and constipating than the latter, it may be administered in large doses without causing any symptoms of intolerance or toxity, and it is not followed by any ill after-effects. Huchard speaks of salicylate of magnesium as having rendered him wonderful services in the treatment of typhoid fever; the ataxic symptoms disappeared, the fetor of the breath vanished, the distended abdomen was diminished in size, and the foul odor of the stools was banished. He believes that the death-rate from ileo-typhus can be greatly lessened by the timely employment of salicylate of magnesium. The dose may be fixed at 10—15 grains, three times daily.—*Notes on New Remedies.*

**Ulexine.**—Ulexine is an alkaloid derived from the seas of Genista, or common gorse. It is crystaline in form, has a bitter taste and is soluble in water. In medicinal doses ulexine first acts as a stimulant, and then as a depressant of the respiratory mechanism; in larger doses it paralizes respiration, slows and weakens the pulse, and finally causes narcosis through its influence on the nervous system, the muscles retaining their electric excitability till death. It also has a powerful effect on the kidneys, causing constriction, followed by a very large expansion, of short duration. Ulexine is a more powerful diuretic than Sparteine, or preparations of Sarothammus scoparius, and has been used with great success in cases of dropsy due to heart disease. As an antidote to strychnia, it not only prevents the onset of the strychnia convulsion, but has the power of checking them after they appear. The dose varies $\frac{1}{20}$—$\frac{1}{10}$ of a grain. The Liquor Ulex Diureticus is the only preparation to be had so far.—*Ib.*

**Kandol. (Candahol, Kanadol.)**—Dr. Njuschkon advises the use of Kandol in place of ether or cocaine. It is a product formed by the distillation of naphtha, and is a perfectly clear, colorless, extremely volatile liquid, burning readily, and having an odor similar to that of benzine. It can be mixed with a small quantity of water or alcohol. It is used in the form of a spray as a local anæsthetic, and in one minute will reduce the temperature to 10° C., and keep it uniformly so for some time. The skin becomes hard, and completely anæsthetized; there is no bleeding at all, or the blood coagulates as soon as it makes its appearance, so that all operations may be performed with great ease and rapidity. Kandol, although well discussed in pharmaceuticas circles of late, is not yet for sale.—*Ib.*

**How to Distinguish Antifebrin from Phenacetin.**—Antifebrin and phenacetin are sufficiently alike externally and of such different values pecuniarily that it is possible that an endeavor might be made to adulterate one with the other. It is, therefore, well to know the chemical reactions distinguishing them. These, according to Schwarz and Ritsert, are as follows: 1. One gramme of phenacetin is heated with two centimetres of solution of caustic soda, and a few crystals of chloral hydrate are added. If the phenacetin is pure, an agreeable aromatic odor is evolved; if antifebrin is present, the odor is extremely disagreeable, and due to the production of phenylcarbamine. 2. The sample is boiled with solution of caustic soda; in this circumstance antifebrin produces analine, which floats upon the surface of the liquid in the form of oily drops; this does not occur with phenacetin. By shaking up the liquid with ether, evaporating the latter, and adding a little water, and a drop of liquid phenol, and a little of a filtered solution at 10 per cent. of hypochlorite of lime, a greenish-blue color is obtained if antifebrin is present. Under like conditions perfectly pure phenacetin gives a cherry-red solution, which is not changed on addition of either hydrochloric acid or ammonia.—*London Lancet.*

**Contraindications for the use of Antipyrin during the Menstrual Period.**—Dr. H. Huchard states that a year ago he administered 15 grains of antipyrin to a woman suffering from violent dysmenorrhœa. As the result of the administration of this drug, the menstrual flow was suddenly arrested. The patient was seized with violent chill, chattering of the teeth, the face became cyanosed, and there were frequent attacks of syncope, the pulse was small and weak, and the patient complained of great headache. The condition was such as to cause great anxiety for nearly an hour, when the effects gradually passed off. Dr. Huchard thinks that he has in two other cases observed similar symptoms, although less marked, and he now regards the presence of the catamenial flow as a positive contra-indication to the use of antipyrin.—*Therapeutic Gazette.*

**Gonorrhœal Rheumatism.**—White brought before the class a female patient for the purpose of showing the difference between ordinary rheumatism and that due to gonorrhœal infection. He said:

"Gentlemen, in this case it is interesting to know whether we are dealing with a case of acute rheumatism of idiopathic origin or one due to joint infection from gonorrhœa. There is a history of gonorrhœa three weeks ago, followed by enlargement of the right wrist-joint, which is painful and tender on pressure. There has been no rise in temperature, no acid, strong smelling perspiration and no evidence of cardiac affection. There is no personal or family history of previous rheumatism. If a patient comes to you and says, 'My knee or ankle or wrist-joints are swollen,' and you find there is but slight elevation of temperature, that the urine is but slightly altered, and that there is no acid perspiration, you may be safe in dealing with it as a case of gonorrhœal rheumatism, especially if there is a history of previous gonorrhœa. In rare cases, however, gonorrhœa and acute rheumatism may co-exist, which would render the diagnosis difficult. I have no hesitation in pronouncing this case one of gonorrhœal rheumatism, which is about the most troublesome joint affection you can be called upon to treat. That it is a mild form of pyæmia is the theory now most accepted. The pathology of the disease seems to be that it is due to the absorption of pus into the system from the urethra or vagina. It is much more frequent in the male than in the female—the proportion being about ten to one; its prevalence in the former being explained (if we accept the pyæmic theory) by the fact that the long urethra presents a greater surface for absorption of the purulent secretion.

"With regard to treatment, it may be laid down as a general rule that the iodides and salicylates are of very little use. The plan I have adopted is to give large doses of sulphate of quinine—say five grains, six times a day during the acute stage. This I follow with small doses of the mercurials—1-24 gr. of the bichloride or ⅓ of a grain of the proto-iodide of mercury. The affected joint is placed in a fixed splint and wrapped in cotton wool. I have had the most excellent results from this line of treatment in a very large number of cases."—*Medical Times.*

**Treatment of Baldness.**—Dr. Lassar (Therap. Ztsch., December, 1888) recommends for alopecia areata the following treatment: For the first six or eight weeks an experienced hand should thoroughly soap the scalp for ten minutes daily, using for this a strong tar soap. A good lather having been formed, it should be removed with an irrigator, using first lukewarm and then cold water. The cold douching will, after several repetitions, harden the scalp somewhat and prevent catching cold. After the scalp has been dried thoroughly the following lotion should be applied:

℞ Solution of bichloride of mercury, 8 grs. to 5 oz.
Glycerin.
Cologne water, ää ℥ ij ʒ v.

Then the scalp should be rubbed dry with alcohol, ninety per cent., to which one-half per cent. of naphthol has been added. The scalp now being free from any fat whatever, the following is applied:

℞ Salicylic acid, 30 grains.
Tincture benzoin, 45 grains.
Neat's foot oil, ℥ iij ʒ ij.

M. This treatment carried out daily for some weeks will be followed with good results. Dandruff and itching will disappear. Hairs which are stiff and dry, become flexible and oily, and where no hair was, hundreds of small hairs will make their appearance. Of course this only holds good when the hair-growth is not destroyed.—*Ex.*

**A New Treatment of Erysipelas.**—Dr. F. P. Henry, in an article on Antiseptic Medication published in Med. News, says: I have lately been treating erysipelas with hypodermic injections of the bicyanide of mercury. My reason for employing this salt in preference to corrosive sublimate is that the former is compatible with cocaine while the latter is not. A hypodermatic injection of corrosive sublimate is so painful that few will consent to its repetition, while the bicyanide, as I have employed it, is comparatively painless. The following formula is the one I am accustomed to use:

℞ Hydrarg. bicyanid, grs. ij.
Cocaine hydrochlorat, grs. iv.
Aquæ destillat., ℥ ss.

M. Fifteen minims to be injected beneath the skin.

I have made about two hundred injections of this mixture in cases of syphilis and erysipelas, always using the dose above mentioned, and have never seen any untoward constitutional symptoms from it, beyond a momentary faintness, which I altributed to the cocaine. Several of the patients were delicate women.

I have treated ten cases of erysipelas capitis with injections of the bicyanide, and without entering into details I may state that when the disease is beginning, for example, at the side of the nose and extending toward one or both cheeks, this treatment tends to abort it. It is of great importance that the injection be made, not into the inflamed patch, but a little beyond its border.—*Med. Age.*

**Urine of Business Men.**—There is no question that too little attention is paid to urinalysis, by the majority of practicing physicians, especially the microscopic part of the work. All know how to test for albumen and sugar, but very few can make a quantitive analysis of urea, and a still smaller number have no microscope, neither do they know the most common crystals. An examination of the urine will reveal the incipiency of many diseases which can be throttled at the outset, and then the physician can exercise his prophylactic powers, the most useful part of his life work, for it is better to prevent than to cure.

Dr. Clifford Mitchell, of Chicago, in the *Medical Era* seems to have been especially interested in the urine of business men, and he emphasizes some important points.

The urine of the over-worked and over-fed American business man usually shows in the beginning, before any renal disease is actually present the following characteristics:

The total quantity of the urine for the twenty-four hours is greatly reduced, sometimes to half the normal quantity. The color is usually, therefore, darker than normal, and there is an abundant sediment in which urates, uric acid, and often calcium oxalate may be seen with the miroscope. The urine being diminished in quantity, and the acidity relatively increased, the urates and uric acid can not be held in solution as they ought to be. Estimation of the total quantity of urea will almost invariably show this substance to be diminished to a figure considerably below normal. Perceptible traces of albumen and sugar I occasionally find in the urine of hyperactic business men, but in many cases both these constituents are as yet absent. I say, "as yet" for sometimes, if all warnings are unheeded, one or the other of these unwelcome visitors, in time, may make its appearance. I regard persistent decrease in both fluids and solids of the urine of great significance in those who eat heartily and exercise little. It is not necessary that either albumen or sugar be present—there is enough trouble without them. When a man of average weight voids daily no more urea than we find in the urine of a delicate woman, or a bedridden patient, it is time to cry "halt." I have found these patients with diminished urea and crystalline sediments, have all sorts of aches and pains. Headache is a very common concomitant. Sleeplessness is is another. If I could have my own way, I would estimate the urea in the urine of every patient in the country suffering from insomnia, or restless unrefreshing sleep. Professor Dowling, in his paper on Lithæmia, has commented on the same thing. Not all insomnia is due to lithæmia, but many cases, apparently hopeless, could be helped were the conditions properly understood.—*Saint Joseph Med. Rec.*

**Perforative Peritonitis Caused by Ascarides Lumbricoides.**—Dr. Murray, in the *Lancet*, reports three cases. They all had symptoms of peritonitis. Post-mortem examination showed perforation of the intestine and round worms in the peritoneal cavity. The perforation was not the result of ulcerated disease in at least one of the cases

In this case the mucous membrane of the alimentary canal was throughout healthy, and presented no trace of tubercular or other disease. In this case, then, death was undoubtedly caused by the worm perforating the bowel.

It is questioned by writers whether the round worm can perforate the intestine. It is generally held that, when found in the peritoneal cavity, it has simply passed thither through an accidental opening. As a result of the author's own observation in Calcutta, he has no doubt whatever that the round worm is capable of causing perforation of the bowel, and actually boring its way into the peritoneum.—*Archives Pedatrics.*

**On the Treatment of the Morphine Habit.**—Instances are very few where the confirmed victim of the morphine habit by will power alone has succeeded in emancipating himself from his bondage. The drunkard has now and then forsaken his cups, and the tobacco victim his pipe or cigar, never again resuming the baneful habit; and all by the strength of a will which, once asserting itself, is never again overcome, despite temptations and the intense craving for an accustomed stimulus. But the unhappy morphinist, when once brought to view with horror the abyss of ruin in which he is sinking, is not likely to escape without extraneous aid. This is due to three reasons: First, the strength of the passion for the narcotic, which for a time becomes increasingly imperious every day; second, the demoralized condition of the individual, whose finer sensibilities and whose better nature have been impaired by the long continued action of the poison on the cells of the cerebral cortex; third, the feeble tension of the will power, or more properly speaking, of those higher energies which represent action inspired by superior motives. Hence, the morphiomaniac can seldom hold out long in the struggle against temptation, and he is in need of the help which the co-operation of friends and medical assistants can give him. There will be a time, even, when restraint will be necessary, as during the first fortnight of the withdrawal, and hence it is not only desirable, but practically essential, that the morphine habitue who would be cured should surrender for a time his liberty to the guardianship and care of persons thoroughly competent to treat his disease; and it is only within the walls of a properly equiped institute that the withdrawal treatment can be carried out.

One disadvantage of the gradual method (alluded to by ERLENMEYER) is the long time during which the patient must remain in the institute. The principle of the treatment being "progressively to diminish the daily quantity of morphine injected, to act slowly, and sometimes to decrease only by several milligrammes a day," months are often required to thoroughly wean the patient from morphine. The treatment by the gradual method is therefore both expensive and painful; the sum of the patient's sufferings is spread over weeks, or even months, instead of being concentrated into a few days, after which the distress is principally over, as in ERLENMEYER'S rapid method.

It is needless to say that the gradual or "tapering off" method cannot be effectively carried out at home, or in a private boarding house. No patient was ever yet weaned from morphine in that way; at least, no inveterate case was ever so cured.

One great obstacle is the facility with which the patient can cheat the physician and obtain clandestinely his supply of morphine, and this difficulty, as ERLENMEYER says, even attends the attempt to carry out the gradual method of withdrawal in a private institute.

As an illustration of ERLENMEYER'S manner of withdrawal—his so-called rapid method—we will take a case which he has reported, viz., that of a physician aged twenty-nine years, who had been made a morphinist by repeated attacks of supra-orbital neuralgia. This gentleman had been using one gramme (fifteen grains) of morphine daily subcutaneously.

On the first day of the treatment, the supply of morphine was reduced to 0.33 (five grains). On the second day, the supply was still further cut down to 0.12 (two grains). On the third day, it was reduced to 0.09 (one and a half grains). On the fourth day, the morphine was reduced to 0.06 (one grain). This was all the morphine that was given hypodermically. A full dose of laudanum (25 drops) was administered on the sixth day on account of diarrhœa. Alcoholic stimulants, bromides, chloral, concentrated broths, milk, etc., were given according to indication; in ten days time the patient was weaned.

A patient that has been using daily quantities of 0.5 to 0.6 (seven and one-half to nine grains), is on the first day of the treatment cut down to two decigrammes (three grains). The second day the morphine is reduced to one decigramme (one and one-half grains). On the third day the same amount is given. On the fourth day 0.5 (three-fourths of a grain) suffices. On the fifth day, there is necessity for 0.06 (one grain). On the sixth day 0.03 (one-half grain) is administered; on the seventh day 0.01 (one-sixth grain); after this, the morphine is discontinued altogether. The supervention of diarrhœa requires an occasional dose of laudanum.

Such, in brief, is ERLENMEYER'S method of treatment which is the result of fifteen years experience and study, and which under the supervision of this German authority and in his institute has been eminently sucsessful.—*Medical Age.*

**Treatment of Ingrowing Toe-Nail.**—Dr. Theodore Clemens, of Frankport, strongly recommends the employment of tinfoil in the treatment of ingrowing toe-nail. He first has the toe thoroughiy washed with soap and carefully dried. He then envelopes the whole nail with tinfoil, putting a strip between the portion that grows in and the raw surface caused by it. The tinfoil is fixed by means of a very thin layer of common wax, and the patient told not to wash the part, but to use dry bran for rubbing off the dirt. Of course the toe has to be repeatedly dressed with tinfoil; but, if the operation is carefully performed, it is surprising how long the tinfoil will remain intact, even when the patient is, as was usually the case in Dr. Clemens' hospital practice, very poor and very badly shod. The results are stated to have been most satisfactory, and are ascribed by Dr. Clemens not merely to the mechanical action of the tinfoil, but to the effect of the permanent contact of a combination of metals comprising iron, copper, arsenic, molybdenum, wolfram and bismuth, with a moist and growing portion of flesh. This, he says, brings about in a few weeks the complete healing of the sore, and causes the nail to grow more slowly and in a more healthy manner.—*Lancet.*

**Emptying the Bladder by Manual Compression.**—Dr. Julius Heddaeus, of Idar, has found manual compression of the bladder an admirable substitute for catheterism in a considerable number of cases where mechanical aid of some kind is imperatively called for. He employs two methods, which may be used alternately, so as to give relief to the hands when they get tired. In the first method the surgeon stands by the side of the patient, who lies on his back. Looking toward the patient's face, he lays the right hand on the left, puts the thumbs together, and then grasps the bladder so that the thumbs lie close to the symphysis, pressure being made of course downward and backward, and the thumbs being gradually approximated to the little fingers. In the second method, the back is turned to the patient's face and the bladder grasped with the hands placed with their ulnar borders against Poupart's ligaments, the finger tips meeting over the pubes. In the first method the chief work falls on the thumbs, in the second on the fingers. The latter is especially applicable in cases where the bladder is only partly full. Manual compression must only be employed very lightly in cases where the bladder is overdistended, and where there is any inflammation or pain it is altogether contra-indicated. It is mainly useful where there is paralysis of the muscular coat of the bladder, whether with or without further paralytic affections. When the sphincter is paralyzed, so that incontinence exists, this method may be employed for emptying the bladder after it has partially filled, so as to prevent the constant dribbling. One advantage of the manual method is that it may be safely taught to the patient's attendants in many cases, and thus the bladder may be emptied at stated times, and as frequently as the surgeon thinks requisite.—*London Lancet.*

**Treatment of Dysentery by Naphthalin Enemata.**—Dr. Gingertoff (*Russ. Medits.*) orders seven or eight grains in an ounce ot water for a single enema, and finds that this in a very short time relieves the tenesmus and the burning sensation at the anus. The patient is able to obtain rest and sleep, and in some cases is cured without any repetition of the enema. In other cases two or three enemata have to be given at intervals of a few hours. Where needful, this treatment is combined with the internal administration of quinine.—*Ex.*

